# An Analysis of the Impact of Sexual Orientation on Longitudinal Depression

**DOI:** 10.1093/geroni/igaa057.3314

**Published:** 2020-12-16

**Authors:** Anna Thompson, Britney Wardecker

**Affiliations:** The Pennsylvania State University, University Park, Pennsylvania, United States

## Abstract

Research suggests that mental health and well-being improve as we age, and this trend is dubbed “the paradox of aging” (Charles & Carstensen, 2010). However, little is known about whether this trend happens for individuals who may experience lifelong disadvantage, such as those who identify as lesbian, gay, or bisexual. We used data from the Midlife in the United States Study (MIDUS) to examine lesbian/gay, bisexual, and heterosexual adults’ changes in depression from 1995 to 2014. Participants identified as lesbian/gay (n = 46), bisexual (n = 37), and heterosexual (n = 3030) and 45.1% identified as female. Participants’ ages ranged from 20-74 years (M = 45.61, SD = 11.41) in 1995 and 39-93 years (M = 63.64, SD = 11.35) in 2014. We analyzed our data using a repeated measures ANOVA and our results indicate that depression decreased on average from 1995 to 2014 for heterosexual [Wilk’s Lamda = .996, F (1, 3029) = 12.23, p < .001] and lesbian/gay adults [Wilk’s Lamda = .848, F (1, 45) = 8.08, p = .007]. However, bisexual adults did not experience this decrease in depression [Wilk’s Lamda = .990, F (1, 36) = 0.36, p = .550] and their depression remained relatively stable. Our results are consistent with previous studies that indicate bisexuals experience poorer mental health when compared to lesbian/gay and heterosexual adults (Bostwick, Hughes, & Everett, 2015). The current research highlights depression as a condition that may not decrease universally over time. We discuss implications for bisexuals’ health and well-being.

